# Treatment of Infantile Convulsions

**Published:** 1900-10

**Authors:** J. W. P. Smithwick

**Affiliations:** LaGrange, N. C.


### TREATMENT OF INFANTILE CONVULSIONS.

By J. W. P. SMITHWICK, M.D.,
LaGrange, N. C.

It is in the treatment of small things, sometimes, that we receive the greatest praise, and so it is usually in the successful treatment of a case of infantile convulsions. When we are called we always find the family in a state of more or less chaos. Nothing so unnerves the female side of creation as for the baby to have convulsions. They almost grow frantic, but we must keep cool and collected and do our duty, which, if well done, will receive everlasting praise, but if not condemnation will be ours. It is my custom, on being called to attend such a case, to give a small, say half an ounce of tepid water containing from ten to thirty drops of bromidia, amount to be regulated according to age of patient, as an enema, and to order a hot mustard bath at once, immersing the whole body beneath water and gently applying friction. This bathing is keep up for about fifteen minutes, when the patient is removed and wrapped in a blanket to induce a perspiration. By the time the bath is finished the whole muscular system will be relaxed and the infant will drop off into a sweet, natural and refreshing sleep, and if it receives the proper treatment, from this on will have no more convulsions. At this stage I make as thorough examination as I can without disturbing the sleeper, and get all the information from the mother, which is never to be neglected. Sometimes the convulsions will be due to digestive disturbances, and at others, to fever, etc. The cause should be ascertained in each individual case, as otherwise we cannot intelligently direct our treatment. I have frequently had the little fellows, after administering the above detailed preliminary treatment, evince symptoms of returning spasms, but when the intestinal tract was thoroughly cleansed with minute doses of calomel or gray powder these symptoms would all disappear. It is often the case with young children that an attack of an acute disease will be ushered in with a convulsion. In these cases the true nature of the disease is to be recognized and treated as such. If this be the case I always leave some bromidia, and direct it to be given in appropriate doses as the conditions and symptoms seem to demand, and I have always had good results from its use in this class of cases. It quiets all excitement of the nerves, overcomes wakefulness, and causes the patient to rest in a quiet and passive state, and to be able to sleep naturally. I give a few clinical notes, as follows:

Was called to visit an infant ten months of age. Found it just recovering from a convulsive seizure, which was the third one that day. I immediately gave it five minims of bromidia in a tablespoon of warm water by the rectum, and placed it in a tepid mustard bath up to its neck. Gentle friction was applied for ten minutes, when it was removed from the bath and wrapped in a blanket. In a few minutes it was bathed in perspiration and sleeping very quietly, and breathing quite naturally, the first time, the mother informed me, since the first convulsion. The mother gave the history that it had "sour stomach" the previous night and vomited that morning, and that it seemed to have some fever. I began the administration of calomel in one-tenth grain doses every half-hour until ten should be administered. The next day I called and found the infant as well as ever as far as could be determined, and it had no more trouble at that attack.

Was hurriedly called to see a child two years of age one evening, and found it in the midst of a convulsion. I immediately placed it in a warm mustard bath of about 110 F., and as soon as the convulsive seizure passed off gave it twenty minims of bromidia in a tablespoon of water by the rectum. The child was wrapped in a blanket and placed in bed, and soon was sleeping naturally. I obtained the history that he seemed to have some fever all the previous morning, and was very much indisposed, which was noticed by its mother, as he had always been playful unless sick. His temperature was 103.5 F., soon after he went to sleep, but it soon began to fall and went to almost normal in three hours' time. Fearing a return of the convulsions I left some bromidia to be given five drops at a dose if he manifested undue nervousness or evinced signs of returning convulsions. The next day I found him with a temperature of 100.5 F., and his mother informed me that she noticed that he felt cool and yawned several times early that morning. I began the administration of antiperiodic remedies, and gave instructions about the administration of bromidia if it was necessary. I called the next day in the afternoon, and he had a temperatue of 99 F., and was improving rapidly. His mother was compelled to give only one dose of the bromidia the previous day, and since that time he has rested and slept well.

It will be noted that in these cases the administration of bromidia had a very happy effect in preventing a recurrence of the convulsions. Its formula is admirably adapted to such cases, containing as it does chloral, bromide of potassium, hyoscyamus and cannabis indica, which I have found far more effective if given in the above combination than if given alone.

